# Effectiveness of the Maternal and Child Health handbook in Burundi for increasing notification of birth at health facilities and postnatal care uptake

**DOI:** 10.1080/16549716.2017.1297604

**Published:** 2017-05-02

**Authors:** Kayo Kaneko, Jacques Niyonkuru, Ndereye Juma, Térence Mbonabuca, Keiko Osaki, Atsuko Aoyama

**Affiliations:** ^a^ Department of Public Health and Health Systems, Nagoya University Graduate School of Medicine, Nagoya, Japan; ^b^ Direction of Healthcare Supply and Demand, Ministry of Health and Fight against HIV/AIDS, Bujumbura, Burundi; ^c^ National Reproductive Health Program, Ministry of Health and Fight against HIV/AIDS, Bujumbura, Burundi; ^d^ Direction of the Internal Administration, Ministry of Home Affairs, Bujumbura, Burundi; ^e^ Human Development Department, Japan International Cooperation Agency, Tokyo, Japan

**Keywords:** Maternal, newborn and child health, home-based records, birth certificate, post-natal care, Africa

## Abstract

**Background**: In Burundi, birth certificate ownership (56.4%) and postnatal care (PNC) coverage (30%) remain low. Birth certificates prove birth registration and allow clients to receive free medical care including PNC. To obtain birth certificates, notification of birth by witnesses is indispensable. However, use of existing parallel home-based records for mother and child has prevented clients from successfully receiving notification of birth and related information.

**Objective**: To assess the effectiveness of the Maternal and Child Health (MCH) handbook for increasing notification of birth at health facilities and PNC uptake.

**Methods**: Pre- and post-introduction measurement were applied including: (i) structured interviews with two different sets of randomly selected mothers having infants aged less than six weeks at the pre- or post-studies; and (ii) secondary data from the national health management information system.

**Results**: 95.1% of mothers had an MCH handbook post-study. Significant improvement was observed in the proportion of mothers receiving notification of birth at health facilities, from 4.6% to 61.0% (95% confidence interval [CI]: 55.9%–66.2%), and the proportion of mothers receiving guidance on PNC, from 35.9% to 64.2% (95% CI: 59.2%–69.3%). The annual PNC coverage (43.9% to 54.2%; *p* < 0.05) in the Gitega District significantly increased from 2013 to 2014. Among MCH handbook owners, mothers giving birth at hospitals/clinics had 2.62 higher odds (95% CI: 1.63–4.22) of obtaining notification of birth than mothers giving birth at health centers. Conversely, mothers delivering at hospitals/clinics had 0.51 lower odds (95% CI: 1.63–4.22) of receiving PNC guidance than mothers delivering at health centers.

**Conclusions**: As previous studies showed, the MCH handbook appeared to help health personnel provide guidance on PNC, thereby it may have increased PNC. Furthermore, this study suggests the handbook contributed to every birth being counted. However, to increase the effectiveness of the handbook, health personnel should be encouraged toward its proper use.

## Background

Burundi is a landlocked country in Eastern Africa (population: 9,850,000, area: 27,800 km^2^). A 12-year sociopolitical crisis significantly delayed the country’s socioeconomic development. The maternal mortality ratio and under-five mortality rate were 712 per 100,000 live births and 82 per 1000 live births, respectively, as of 2015 [1]. Since 2006, free medical care for pregnant women and children under five has been introduced and has improved coverage of antenatal care (ANC), delivery assisted by skilled birth attendants, and child vaccination [2]. Nevertheless, birth certificate ownership (56.4%) and postnatal care (PNC) coverage (30%) remain low [2].

A birth certificate is an important document that proves birth registration and allows clients access to essential services such as free medical care including PNC and school admission [3]. To obtain a birth certificate, notification of birth including basic birth information such as the birth date, birth weight, name of mother, birthplace, and name of birth attendants provided by witnesses (birth attendants, local government official or health institution) is indispensable [3]. However, use of existing parallel home-based records for mother and child has prevented clients from successfully receiving notification of birth and records around birth at health facilities. Before this pilot study, there were four types of home-based records: the ANC card, maternal tetanus vaccination card, vaccination card for children, and the child health care handbook. As the first step to address these problems, the Burundian Ministry of Health and Fight against HIV/AIDS (MOH), the Ministry of Home Affairs, and the Japan International Cooperation Agency (JICA) collaborated with relevant partners to develop and introduce the Maternal and Child Health (MCH) handbook, which contains a page for notification of birth.

The MCH handbook, an integrated home-based record, consists of records along the continuum of care for mothers and children, such as ANC, delivery, PNC, vaccination, child growth monitoring, and clinical visits. Moreover, the MCH handbook contains educational messages to raise the demand and awareness of healthy behavior of women in pregnancy and motherhood, and improve their essential service utilization [4–8].

Previous studies have shown that mothers and children who acquire a notification of birth are more likely to attend PNC [9–11]. MCH handbooks had already been introduced in several countries; however, to our knowledge, no studies have assessed their effectiveness on improving notification of birth ownership. Thus, we describe here a pilot implementation of the Burundian MCH handbook, which contains a page for notification of birth.

This paper aims to assess the effectiveness of the MCH handbook in increasing notification of birth ownership and PNC uptake in all 23 health facilities in the Gitega District, Burundi.

## Methods

### Study design

Pre- and post-introduction measurement were applied including: (i) structured interviews with two different sets of randomly selected mothers having infants aged less than six weeks at the pre- or post-studies and (ii) secondary data from the national health management information system.

We set socio-demographic status (i.e. age of mother, parity, age of infant, educational level) and delivery place by type of health facility as confounding variables, the proportion of mothers having the MCH handbook, having received notification of birth at a health facility, having delivery mode records, having accurate birth weight data by recall or records, and receiving guidance on PNC as outcome variables, and the annual PNC coverage as the outcome indicator.

### Local setting

This study was conducted in the Gitega District (population: 256,730) [12] in the mountainous area of central Burundi about 100 km from the capital, Bujumbura. The Gitega District is one of four Districts (i.e. Gitega, Kibuye, Mutaho, and Ryansoro) of the Gitega Province that has 2 administrative offices for birth registration and 23 health facilities, that is, 2 referral hospitals, 1 clinic, and 20 health centers. The MCH handbook was piloted in all 23 health facilities providing maternal and child health care services in the Gitega District (Table 1). The estimated annual birth number in the Gitega District was 12,066, 67.4% (8136 births) of which occurred in health facilities [12].

**Table 1.** Stratified sample size along with the number of births in each facility for the estimated sample size of 368 with 5% excess.

	Name of health facility	Annual birth number in 2011	Stratified sample size in each facility for the estimated sample size of 368 plus 5% excess	Type of health facility
1	Gitega Hospital	2385	106	Hospital
2	Kibimba Hospital	1808	53	Hospital
3	Songa Clinic	422	21	Clinic
4	Vision santé Health Center	0	0	Health Center
5	Bukinga Health Center	219	15	Health Center
6	Gasunu Health Center	351	18	Health Center
7	Gisuru/Giheta Health Center	625	36	Health Center
8	Murayi Health Center	754	40	Health Center
9	Kibimba Health Center	0	0	Health Center
10	ABUBEF Gitega Health Center	0	0	Health Center
11	Cabinet Medical Nduwumwami Health Center	0	0	Health Center
12	Gitega Health Center	0	0	Health Center
13	Mubuga Health Center	506	29	Health Center
14	Mushasha Health Center	111	6	Health Center
15	La Sagesse Health Center	0	0	Health Center
16	S.O.S Health Center	0	0	Health Center
17	Nyamugari Health Center	0	0	Health Center
18	C.M.M Health Center	0	0	Health Center
19	Notre dame d’afrique Health Center	0	0	Health Center
20	Ceru Health Center	65	5	Health Center
21	M.M.K Health Center	224	15	Health Center
22	Rutegama Health Center	223	11	Health Center
23	Rutoki Health Center	493	29	Health Center
	Total	8136	384	

### Sampling framework

To standardize the study participants in the structured interviews, we focused on mothers with infants aged less than six weeks and living within the jurisdiction of each health facility. Study participants were selected randomly from maternity registers, which are compiled at each facility. The sample size for the interview was calculated using the formula for single proportion with the following assumptions: α (type I error) of 0.05, 1–β (power) of 0.60, precision of 0.05, and the proportion of mothers who recorded birth weight data in the latest Demographic Health Survey was estimated to be 60% [2]. We arrived at an estimated sample size of 368 that was proportionally allocated with 5% excess among 23 facilities following the annual number of births in each facility (Table 1).

### MCH handbook introduction process

In the development process of the pilot version of the MCH handbook, all relevant stakeholders (i.e. national programs including reproductive health, immunization, and nutrition, Ministry of Home Affairs, and development partners) participated in several discussions, considering future nationwide scaling-up of the handbook. Table 2 summarizes the components of and main features of the handbook. The contents, design, and layout of the MCH handbook were adjusted and customized according to relevant existing tools and it was tested and finalized with local users. An A5 format with 20 pages was adopted, with a unit cost of less than 0.5 USD to ensure financial sustainability.

**Table 2.** Burundian Maternal and Child Health handbook’s components and features.

Domain	Components	Main features and guiding principles to develop the components
Records of care	Names, identification numbers, and contacts of mother, child, and father/family.	To devise simpler and educative recording field for health providers.
	Vaccination of mother.	The formulation followed old home-based records and existing facility-based records, and all record fields were widened.
	ANC, delivery, PNC, and child growth monitoring.	The record items were updated according to current national health care norms and national health statistic report components.
	Child vaccinations until 5 years old.	
	‘Clinical visits for mother and child.	
	Family planning.	
Notification of birth	Birth date, birth weight, name of mother, birthplace/facility, and name of birth attendants/profession.	The items were made to be consistent with the requirements for civil registration procedures.
	Affixed seal of the health facility.	Health providers are requested to complete the section only in the case of delivery at the health facility.
Educational messages	PNC, birth registration, family planning, I.M.CI (Integrated Management of Childhood Illness), child nutrition, and child growth.	Minimum messages were selected according to the priorities of national health policy.
		Some accompanying illustrations should be included along with messages in the local language, in consideration of the rural literacy rate (76.9%)[2].
		The illustrations were taken from community health workers’ materials, so that community health workers would be able to explain the contents to mothers with enough confidence.

**Figure 1.**
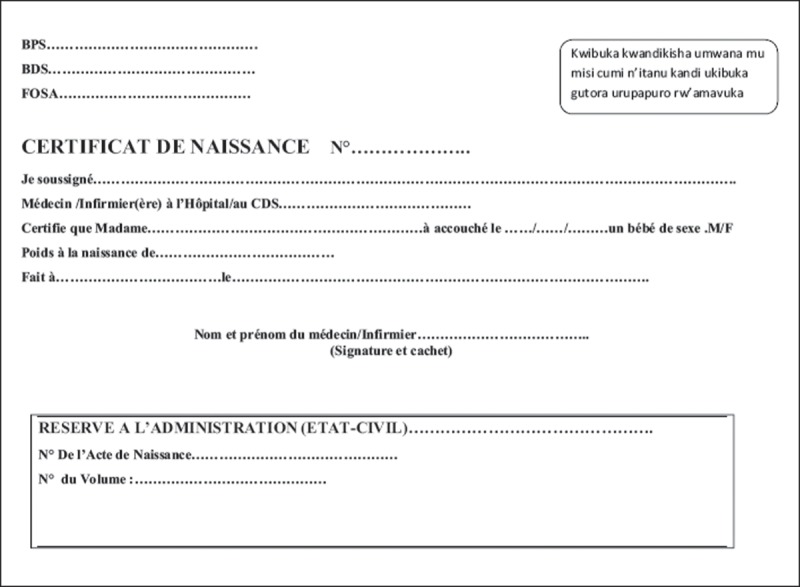
Page for notification of birth in the MCH handbook.

Before introducing the handbook, MCH care providers were trained in the recording method, the educational message, and logistics management of the MCH handbook. Furthermore, all birth registration officers of both of the administrative offices were also officially informed that the pilot version of the handbook contained a page for notification of birth (Figure 1), which was to be referred to at the birth registration points.

Public announcement of the initiation of the MCH handbook in the pilot area was conducted during four days of the National MCH campaign week in December 2013. Specifically, during this time, the handbook was distributed to pregnant women at each health facility and remote campaign site. After the campaign, the handbook was given to pregnant women who were attending ANC for the first time or to those who had arrived at a health facility for delivery without the handbook. To prevent overlapping use of home-based records, the older records were discontinued with the introduction of the MCH handbook [13]. Following the commencement of the distribution of handbooks, we followed up to check on their appropriate use.

### Data collection

The procedures of the study were reviewed and approved by the research technical committee of the Ministry of Health and Fight against HIV/AIDS, Burundi (REF: 633/329/DGSSLS/2013) in October 2013. Structured interviews in November 2013 and July 2014 were conducted respectively with different sets of respondents with children of the same targeted age. Eight research assistants were trained for eight hours on study objectives, questionnaire contents, and interview techniques including ethical considerations. Researchers collected informed consent and conducted the pre- and post-studies with selected mothers by home visits. Completed questionnaires were checked every day by supervisors to ensure accuracy and completeness.

Secondary data on the annual PNC coverage were obtained from the national health management information system for 2013 [14] and 2014 [15].

### Data analysis

Data were entered into Microsoft *Excel* and analyzed therein and with IBM *SPSS Statistics 24.0*. Descriptive statistics summarized characteristics of the respondents in the pre- and post-studies. Chi-square tests were employed to assess the differences in the proportions of all outcome variables and indicators between the pre- and post-studies. When there were fewer than five cases, Fisher exact tests were employed. Additionally, the proportion and the 95% CI are shown for each outcome variable. To assess the influence of confounding variables on outcome variables, we employed logistic regression models. A *p*-value of less than 0.05 was considered statistically significant.

## Results

Between December 2013 and June 2014, 8786 MCH handbooks were distributed and 101 MCH care providers (6 physicians and 95 nurses) were trained. During the study period, a nationwide health campaign was uniformly conducted in December 2013. To our knowledge, there were no other events except for the MCH handbook introduction that would have influenced the results of this study in the Gitega District.

Total valid responses obtained were 370 and 344 from the pre- and post-studies, respectively. There was no significant difference in the socio-demographic status of respondents who participated in the pre- and post-studies, in terms of age of mothers (*p* = 0.15), parity (*p* = 0.42), and final education level (*p* = 0.07) (Table 3).

**Table 3.** Characteristics of respondents in the pre- and post-studies of the MCH handbook in the Gitega District.

	Pre (n = 370)		Post (n = 344)		
	n	(%)	n	(%)	*p*-value*
Age of mothers					
15–34 years of age	335	(90.5)	317	(92.2)	0.15
> 34 years of age	31	(8.4)	27	(7.8)	
Unknown	4	(1.1)	0	(0.0)	
Parity					
1–2	199	(53.8)	188	(54.7)	0.42
3–4	115	(31.1)	107	(31.1)	
> 4	53	(14.3)	49	(14.2)	
Unknown	3	(0.8)	0	(0.0)	
Educational level					
No school education	165	(44.6)	119	(34.6)	0.07
Primary	157	(42.4)	171	(49.7)	
Secondary	38	(10.3)	38	(11.0)	
High school	8	(2.2)	15	(4.4)	
Graduated	2	(0.5)	1	(0.3)	
Age of infant					
Within 2 weeks after birth	79	(21.4)	62	(18.0)	0.15
3–4 weeks after birth	170	(45.9)	146	(42.4)	
5–6 weeks after birth	121	(32.7)	136	(39.5)	
Delivery place by type of health facility					
Health center	196	(53.0)	177	(51.5)	0.69
Hospital and clinic	174	(47.0)	167	(48.5)	

Notes: Pre- and post-studies included different individuals as respondents. *Chi-square test.

After the MCH handbook introduction, significant improvements were observed in the outcome variables (Table 4).

**Table 4.** Changes in proportion of mothers per outcome variables between the pre- and post-studies in the Gitega District.

	Pre (n = 370)	Post (n = 344)	
Variable	Proportion (95% CI)	Proportion (95% CI)	*p*-value
Having MCH handbook	0.0%	95.1%	< 0.05^b^
	(0.0%–0.0%)	(92.8%–97.3%)	
Having received the notification of birth at health facilities	4.6%	61.0%	< 0.05^a^
	(2.5%–6.7%)	(55.9%–66.2%)	
Having data on delivery mode records at home	0.0%	29.7%	< 0.05^b^
	(0.0%–0.0%)	(24.8%–34.5%)	
Having accurate birth weight data at home by recall-based or record	50.8%	73.5%	< 0.05^a^
	(45.7%–55.7%)	(68.9%–78.2%)	
Receiving guidance on PNC from health personnel	35.9%	64.2%	< 0.05^a^
	(31.1%–40.8%)	(59.2%–69.3%)	

Notes: Pre- and post-studies included different individuals as respondents. CI = Confidence interval. ^a^Chi-square test. ^b^Fisher exact test.

First, 95.1% (95% CI: 92.8%–97.3%) of mothers had the MCH handbook in the post-study. Second, the proportion of mothers who received notification of birth at health facilities significantly increased from 4.6% to 61.0% (95% CI: 55.9%–66.2%). Additionally, the proportion of mothers who had records about delivery mode significantly increased from 0.0% to 29.7% (95% CI: 24.8%–34.5%). Furthermore, the proportion of mothers who had accurate birth weight data by recall or records increased from 50.8% to 73.5% (95% CI: 68.9%–78.2%).

**Figure 2.**
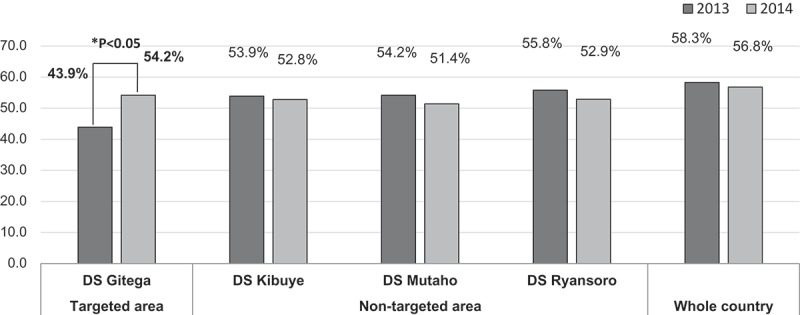
Annual PNC coverage changes from 2013 [14] to 2014 [15] by targeted and non-targeted areas among four Districts (D.S) in the Gitega Province and whole country.

Third, the proportion of mothers who received guidance on PNC from health personnel after delivery increased from 35.9% to 64.2% (95% CI: 59.2%–69.3%); concomitantly, the annual PNC coverage increased from 43.9% [14] in 2013 to 54.2% [15] (*p* < 0.05) in 2014 (Figure 2).

The use of the MCH handbook by health personnel differed according to delivery place. Among MCH handbook owners in the post-study, delivery place as indicated by type of health facility (i.e. health center, hospital, or clinic) influenced the outcome variables after considering potential confounding variables based on respondents’ socio-demographic characteristics. In particular, mothers who gave birth at hospitals/clinics had 2.62 higher odds (95% CI: 1.63–4.22) of obtaining notification of birth, and 3.25 higher odds (95% CI: 1.96–5.39) of having delivery mode records than mothers who gave birth at health centers. Conversely, mothers who delivered at hospitals/clinics had 0.51 lower odds (95% CI: 0.51–0.81) of receiving guidance on PNC than mothers who delivered at health centers (Table 5). Additionally, mothers over 35 years old had 3.03 higher odds (95% CI: 1.01–9.06) of obtaining notification of birth than younger mothers. Furthermore, mothers whose parity was over four had 4.52 higher odds (95% CI: 1.32–15.43) of having accurate birth weight records than mothers whose parity was one or two.

**Table 5.** Association of notification of birth, delivery mode records, birth weight data, and guidance on PNC with socio-demographic characteristics of respondents who received the MCH handbook.

			Notification of birth^a^		Delivery mode records^b^		Birth weight data^c^		Guidance on PNC^d^	
Socio-demographic variable		n	OR	95% CI	OR	95% CI	OR	95% CI	OR	95% CI
Age of mothers	15–34 yr	300	1.00	Ref	1.00	Ref	1.00	Ref	1.00	Ref
	> 34 yr	27	3.03*	1.01–9.06	0.27	0.07–1.09	0.77	0.19–3.09	1.03	0.38–2.80
Parity	1–2	180	1.00	Ref	1.00	Ref	1.00	Ref	1.00	Ref
	3–4	99	1.12	0.66–1.89	0.96	0.54–1.70	1.72	0.97–3.05	0.87	0.51–1.49
	> 4	48	0.74	0.32–1.71	1.22	0.50–2.98	4.52*	1.32–15.43	0.78	0.34–1.77
Age of infant	< 2 weeks	58	1.00	Ref	1.00	Ref	1.00	Ref	1.00	Ref
	3–4 weeks	130	0.87	0.45–1.68	0.65	0.33–1.31	1.34	0.68–2.63	0.69	0.36–1.35
	5–6 weeks	139	1.13	0.58–2.20	0.78	0.39–1.56	2.02	1.00–4.08	0.81	0.41–1.59
Education	No education	115	1.00	Ref	1.00	Ref	1.00	Ref	1.00	Ref
	Primary	163	0.83	0.50–1.39	1.24	0.70–2.20	0.61	0.34–1.10	1.38	0.83–2.30
	Secondary or more	49	1.20	0.56–2.56	1.56	0.72–3.38	0.46	0.21–1.01	2.15	1.00–4.63
Delivery place	Health Center	154	1.00	Ref	1.00	Ref	1.00	Ref	1.00	Ref
	Hospital and clinic	173	2.62*	1.63–4.22	3.25*	1.96–5.39	1.13	0.68–1.87	0.51*	0.32–0.81

Notes: OR: Odds ratio adjusted for other socio-demographic variables in a logistic regression. CI: Confidence interval. Mothers having received notification of birth at health facility, mothers having delivery mode records, mothers having accurate birth weight data by record, mothers receiving guidance on PNC. *p < 0.05.

## Discussion

Consistent with previous studies, which reported that home-based records may help increase health service uptake and promote clients’ health behavior [4–8,16], the Burundian MCH handbook appeared to help health personnel provide guidance on PNC, and thereby may have increased use of PNC. Furthermore, this study revealed the effectiveness of the Burundian MCH handbook in helping to ensure mothers received data around their children’s birth including notification of birth.

The MCH handbook enabled clients to keep their own continuum records, which are essential for effective guidance of care. Both ANC and delivery records inside the handbook could work as guides and references for care afterwards, even for use in maternal and neonatal death surveillance and response. As 58% of children under five years old in Burundi exhibit stunting [2], in particular, birth weight data kept by mothers in the handbook would be vital to accurate child growth monitoring. However, challenges in effective operation remain, as the increase of the proportion of mothers having delivery mode records was limited. Additionally, the post-study showed outcome variables were influenced by type of health facility where deliveries were taken. We need to encourage health personnel to especially record deliveries at health centers and use each piece of information in the handbook for improving quality of care for MCH [17]. According to the association between mother’s socio-demographic characteristics and each outcome variable, to increase the effectiveness of the handbook, we may recommend that health personnel pay attention to younger or low-parity mothers in particular. Furthermore, as correct birth weight is basic information for monitoring of child growth, we need to explore potential barriers for mothers with low parity to acquire correct information on birth weight. This study led MOH and the Ministry of Home Affairs to decide jointly to scale-up use of the MCH handbook nationwide, and to the signing of a ministerial order for its use in birth registration procedures. In developing countries, the overall complexity of the birth registration process commonly presents challenges [3]. Burundi is no exception. Parents who fail to receive their children’s notification of birth at the health facility are required to take three steps for birth registration: (i) to ask the village chief to make a notification of birth attested by three witnesses; (ii) to declare the birth at an administration office using the previously obtained notification; and (iii) to obtain a birth certificate from the administration office. Note that parents who have received notification of birth can skip the aforementioned step (i). This is likely to encourage mothers to proceed smoothly to their children’s birth registration at local administration offices. Closer collaboration between the MOH and civil registration services [18,19] and increasing awareness of the population [20] enable increased birth registration rates in developing countries. Therefore, the MCH handbook, a simple sensitization tool authorized by the joint ministerial order, is expected to improve the birth registration rate so that every child is counted and starts his/her life with social recognition. As Burundi is just at the starting point, we suggest future studies that investigate the relationship between operation of the MCH handbook and birth registration rate and birth certificate possession rate.

## Conclusion

This study assessed the effectiveness of the MCH handbook for increasing notification of birth at health facilities and PNC uptake in the Gitega District, Burundi. As previous studies showed, the MCH handbook appeared to help health personnel provide guidance on PNC, thereby it may have increased the uptake. Furthermore, the MCH handbook ensured that parents possess their child’s notification of birth at a health facility to induce the child’s birth registration with a simpler procedure. This finding suggests that the handbook contributed to ensuring that every birth is counted and every child starts life with social recognition. However, to increase the effectiveness of the handbook, health personnel should be encouraged toward its proper use.

## Paper context

As previous studies have shown, the Maternal and Child Health handbook appeared to help health personnel provide guidance on postnatal care, thereby it may have increased the latter’s uptake. This study revealed the further value of the handbook to help ensure mothers received notification of birth at health facilities, which may contribute to every child starting life with social recognition. However, to increase the handbook’s effectiveness, health personnel should be encouraged toward its proper use.
